# Supplementary material for: Individual prediction tendencies do not generalize across modalities
Source: Psychophysiology. 2023 Sep 10;61(1):e14435. doi: 10.1111/psyp.14435 (PMC10909557; doi:10.1111/psyp.14435)
Supplement: Supplementary file 1 — Data S1. [file PSYP-61-e14435-s001.docx]

# SUPPLEMENTARY MATERIAL

## **Poststimulus decoding of auditory and visual features**

In order to generally verify feature decodability from neural activity and to investigate the duration for which a given stimulus representation remains sustained in both modalities, we conducted a classification analysis that focused solely on trials in the random condition. We trained and tested an LDA classifier in a time-resolved manner from -0.2 s to 1 s using a 5-fold cross validation approach. We find that auditory features (i.e. sound frequencies) can be classified from brain activity starting 0.06 s until 0.67 s following sound onset. Similarly visual features (i.e. gabor patch orientation) can be classified from 0.07 s to 0.74 s after stimulus onset. (Note that in this case “decodability” was defined as the lower boundary of a 95%CI exceeding the chance level of 0.25, which was not corrected for multiple comparisons, since we wanted to gain a liberal estimate for the duration of feature-specific information.) We conclude that auditory as well as visual features can be classified from brain activity starting at ~100 ms until ~700 ms after stimulus onset. Therefore, we argue that 700ms is the time-window in which potential carry-over effects should be controlled for when comparing classifier performance between an ordered (systematic stimulus variation) and a random (unsystematic variation) context.

## **Anticipatory predictions can be found mainly in the auditory modality**

To quantify “prediction tendency”, we compared the classifier's prestimulus tendency for a highly probable forward transition in an ordered context to the same tendency in a random context. Cluster-based permutation showed an overall auditory prediction tendency (i.e. ordered > random) in two prestimulus clusters from -0.23 s to -0.2 s (p = 0.035) and from -0.17 s to -0.07 s (p = 6.9*10-4). For visual prediction tendency, we found only a trend suggesting prestimulus predictions in a short cluster from -0.24 s to -0.22 s (p = 0.056).

Additionally, we conducted a second analysis (see also Demarchi et al., 2019), where we trained the classifier on poststimulus brain activity of random trials and time-generalised its performance to capture potential prestimulus representations in ordered as well as random trials (for more information on the temporal generalisation approach see also King & Dehaene, 2014). One advantage of this approach is that, as the classifier is trained on random trials only, it remains unbiased and does not require for the preceding stimuli to be matched between conditions. Using a cluster-based permutation test, we compared the classifiers performance to generalise from post- into prestimulus intervals between the ordered and the random condition separately for each modality. We found that in the auditory modality, there was a positive prestimulus cluster (p = 1.99*10^-4^) indicating that, in an ordered context, brain activity patterns preceding a stimulus (-0.23 s - -0.05 s) were more similar to pure bottom-up (0.14 s - 0.38 s) processing patterns than in a random context (see **Fig. S3**). We conclude that anticipatory predictions contain feature-specific activations similar to those following sound onset. In the visual modality, however, we found no such anticipatory cluster (see **Fig. S3**). Furthermore we found significant post-stimulus clusters in both modalities left-sided to the diagonal (see **Fig. S3**). This shows that later training time in the random condition genelarised better to earlier training time in the ordered condition, indicating an advanced stimulus processing with stronger statistical regularity. In detail we found 2 significant post-stimulus clusters in the auditory modality: one cluster (p = 0.012) suggesting that later stage processing in the random condition (-0.44 s - 0.5 s) generalises to earlier stages in the ordered condition (0.16 s - 0.26 s) and another cluster left sided to the diagonal (p = 0.020) indicating a latency shift from 0.09 s - 0.18 s in the random to 0.08 s - 0.13 s in the ordered condition. In the visual modality we found a similar latency shift from 0.11 s - 0.24 s in the random condition to 0.1 - 0.2 s in the ordered condition (p = 0.001). This suggests that in both modalities the effect of increased statistical predictability leads to an advanced poststimulus processing. Additionally, in the auditory modality increased regularity is associated with a strong prestimulus anticipation of features. In sum, our findings suggest that, although statistical regularities are learned in both modalities, anticipatory predictions can be found mainly, if not exclusively, in the auditory modality.

# FIGURE CAPTIONS

***Fig. S1:*** *Time-resolved decoding accuracy for auditory and visual features: sound frequency as well as gabor patch orientation can be classified from brain activity from ~100 ms until ~700ms after stimulus onset in a random context (shaded area indicates 95%CI, dashed line shows the chance level of 0.25; N = 35).*

***Fig. S2:*** *Tendency to represent a “forward” transition (compared to a “repetition” transition), separately for different entropy levels and modalities. On a group-level there seems to be an anticipatory tendency to represent stimulus features of high probability (i.e. forward transition) in a predictable context in the auditory modality, but not in the visual modality. (Note that this Figure shows the same data as* ***Fig. 2A****, but with separate lines for each entropy condition. Y-axis represents the classifier dvals for a “forward” transition before subtraction (ordered - random) and the solid lines on x-axis indicate significant time-points (ordered > random); N =35).*

***Fig. S3:*** *Temporal generalisation of feature-specific activations in the auditory (left) and visual (right) modality. Left: In the auditory modality there is a significant difference in the generalisation from post- to prestimulus processing between entropy levels. This suggests that in an ordered, but not in a random, context people generate feature-specific anticipatory predictions, that resemble bottom-up processing. Right: In the visual modality, however, we find no significant evidence for a regularity-dependent generalisation from post- to prestimulus acitvations. (Y-axis represents classifier poststimulus training-time, X-axis represents classifier pre- and poststimulus testing-time and the dashed-grey line indicates the diagonal; T-values are shown in color and marked outlines indicate a significant cluster in the comparison ordered vs. random; N = 35).*

# REFERENCE

King, J.‐R., & Dehaene, S. (2014). Characterizing the dynamics of mental representations: The temporal generalization method. Trends in Cognitive Sciences, 18(4), 203–210. <https://doi.org/10.1016/j.tics.2014.01.002>
