# Supplementary figures and images for: Individual prediction tendencies do not generalize across modalities
Source: Psychophysiology. 2023 Sep 10;61(1):e14435. doi: 10.1111/psyp.14435 (PMC10909557; doi:10.1111/psyp.14435)

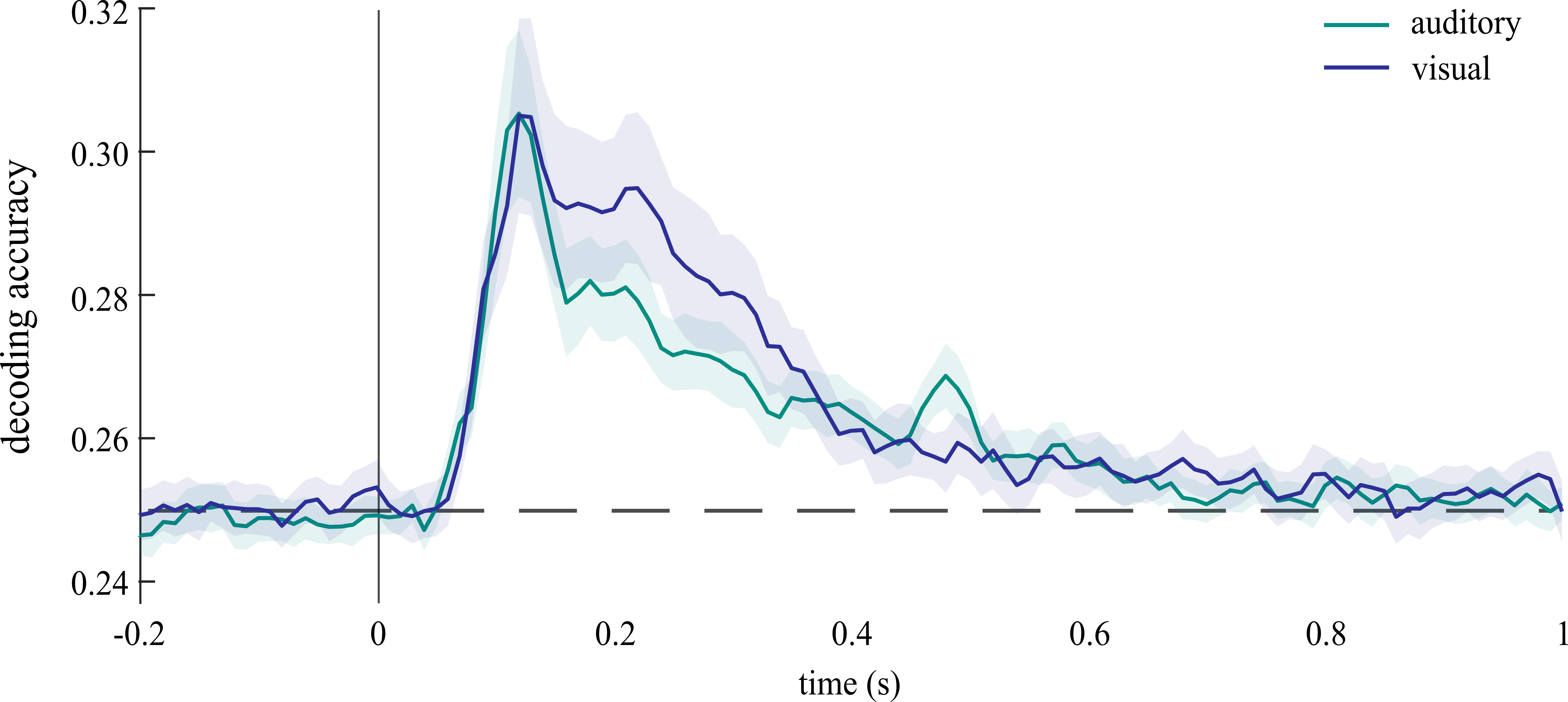

Supplement: Supplementary file 2 — Figure S1. Time‐resolved decoding accuracy for auditory and visual features: sound frequency as well as gabor patch orientation can be classified from brain activity from ~100 ms until ~700 ms after stimulus onset in a random context (shaded area indicates 95%CI, and dashed line shows the chance level of 0.25; N = 35). [file PSYP-61-e14435-s004.png]

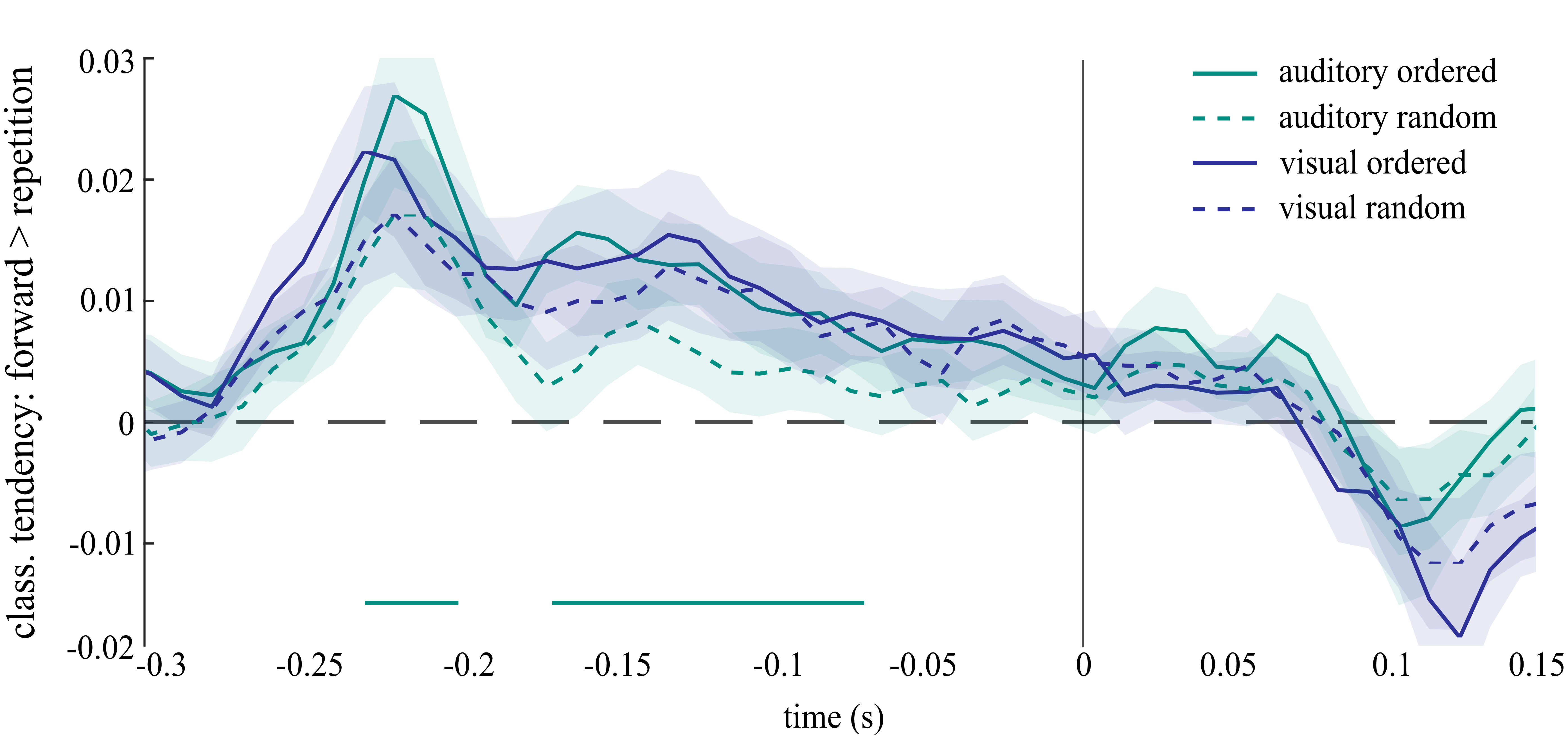

Supplement: Supplementary file 3 — Figure S2. Tendency to represent a “forward” transition (compared to a “repetition” transition), separately for different entropy levels and modalities. On a group‐level, there seems to be an anticipatory tendency to represent stimulus features of high probability (i.e., forward transition) in a predictable context in the auditory modality, but not in the visual modality. (Note that this figure shows the same data as Figure 2a, but with separate lines for each entropy condition. The y‐axis represents the classifier dvals for a “forward” transition before subtraction (ordered − random), and the solid lines on the x‐axis indicate significant time‐points (ordered > random); N = 35). [file PSYP-61-e14435-s002.png]

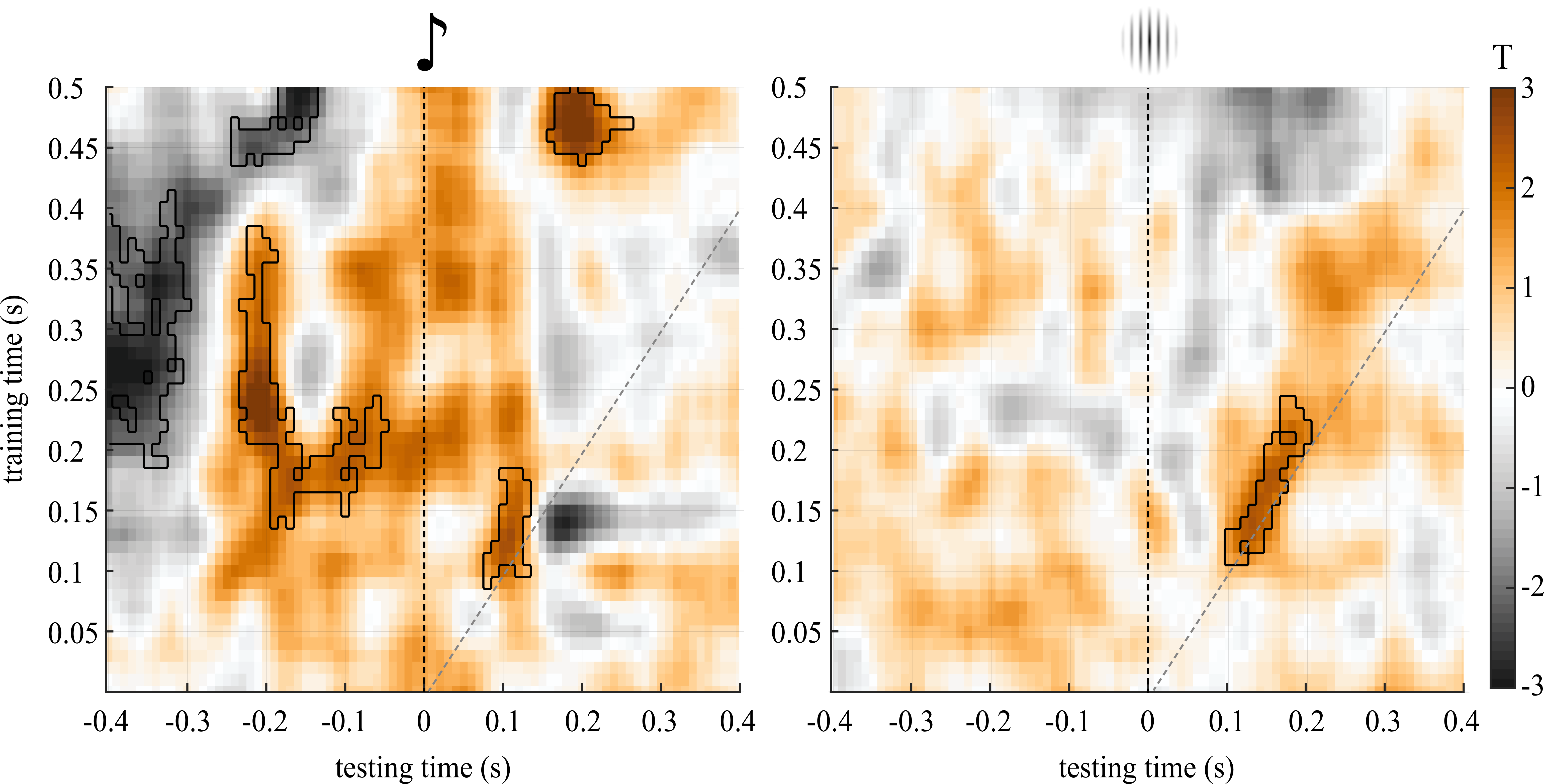

Supplement: Supplementary file 4 — Figure S3. Temporal generalization of feature‐specific activations in the auditory (left) and visual (right) modality. Left: In the auditory modality there is a significant difference in the generalization from post‐ to prestimulus processing between entropy levels. This suggests that in an ordered, but not in a random, context people generate feature‐specific anticipatory predictions, that resemble bottom‐up processing. Right: In the visual modality, however, we find no significant evidence for a regularity‐dependent generalization from post‐ to prestimulus acitvations. (The y‐axis represents classifier poststimulus training‐time, x‐axis represents classifier pre‐ and poststimulus testing‐time and the dashed‐gray line indicates the diagonal; T‐values are shown in color, and marked outlines indicate a significant cluster in the comparison ordered vs. random; N = 35). [file PSYP-61-e14435-s003.png]
